# Editorial: The Second Intercontinental NMR Conference ICONS2021

**DOI:** 10.1007/s00723-021-01320-7

**Published:** 2021-04-17

**Authors:** D. Abergel, G. Buntkowsky, K. Ivanov, P. K. Madhu

**Affiliations:** 1 Laboratoire Des Biomolécules, Département de chimie, École Normale Supérieure, PSL University, CNRS, Sorbonne Université, 75005 Paris, France; 2 Institute of Inorganic and Physical Chemistry, Technical University of Darmstadt, 64287 Darmstadt, Germany; 3 International Tomography Center SB RAS, Novosibirsk, 630090 Russia; 4 TIFR Centre for Interdisciplinary Sciences, Tata Institute of Fundamental Research Hyderabad, 36/P Gopanpally Village, Ranga Reddy District, Hyderabad, 500107 India

**ICONS2021,** organized during February 10–12, 2021, was the second edition of the on-line magnetic resonance conference series called “**I**nter**co**ntinental **N**MR **S**eminar”. The ICONS conferences are an off-shoot of the weekly Intercontinental NMR Seminar Series that started on April 8, 2020. This seminar series enables communication and dissemination of research ideas among the magnetic research community especially in the times of the COVID-19 pandemic. In the weekly series, until now, more than three dozen scientists from five different continents have presented their recent results. The seminar series gives both early-stage and experienced researchers an opportunity to give seminar talks and interact with colleagues from all over the world.

The ICONS2021 conference attracted registrations from nearly 348 people from 34 countries (in the spirit of the meeting, covering 6 continents (Europe, North America, South America, Africa, Australia, Asia) and spanned 17 time zones from Japan over Europe to the West Coast. The meeting talks were broadcast across the Zoom and YouTube platforms. The average combined attendance was around 180.

The ICONS seminar series is open to all areas of magnetic resonance and covers the full range of Magnetic Resonance, i.e. EPR, NMR, MRI, and their various hybrids. While the first ICONS conference in 2020 (see report in APMR [1] for details) was equally broad in scope, the present conference was focused on fields where the interaction of electron and nuclear spins play a pivotal role. Emphasis was given on various flavors of Dynamic Nuclear Polarization. The main goal of this focus was the idea to further support and stimulate the interactions between EPR-oriented and NMR-oriented groups and MR. To achieve this goal, thirteen speakers were selected among the leading experts in these fields and invited to report at the conference.

In this area, the meeting covered a wide range of topics, including the design of stable radicals, technical developments like the application of arbitrary waveform generators controlling microwave pulses, over pulsed EPR and hyperfine spectroscopy, and the spectroscopy of nitrogen-vacancy centers in nanodiamonds, to chemically induced nuclear polarization and DNP applications in MRI.

**Fedor Jelezko**, Ulm, introduced the diamond-based quantum sensing via nitrogen vacancies, as a possible means to study ultralow concentrations of nuclear spins and the NMR detections of a single protein molecule and reported the tremendous sensitivity and spatial resolution gains achievable by this technique.

**Akinori Kagawa**, Osaka, reported recent advances in the field of DNP experiments with optically polarized electron spins by photo-excitation of electronic triplet states. Owing to the selection rules of photoexcitation and inter-system crossing, such excited-triplet states often exhibit much higher electronic spin-polarization than thermally polarized spins and do not depend on cryogenic temperatures. The presentation discussed both the advantages of these states and also the experimental difficulties of the technique and how to overcome some of those to broaden the application profile of the technique.

**Olivier Ouari**, Aix-Marseilles, introduced the current state of the art of the development and synthesis of efficient polarizing radicals in particular for applications in high-field DNP enhanced NMR. He explained in detail the careful adjustment of the molecular and spin parameters, necessary to get an optimized cross-effect at the different magnetic fields, and how this optimization procedure is achieved by a combination of chemical intuition, EPR characterization, quantum chemical calculations, and synthetic screening.

**Bob Griffin**, MIT, first gave an overview about the current state of the art of Dynamic Nuclear Polarization enhanced NMR spectroscopy and the underlying technological developments, which have led to its current success. Then he discussed both the potential and the difficulties of pulsed (also known as time-domain) DNP techniques. While these techniques have the potential to create strong hyperpolarization at high magnetic fields, they are extremely demanding with respect to the necessary micro-wave hardware, as it is very difficult to create the necessary high-power microwave pulse close to the THz regime. However, with high-frequency gyro-amplifiers, there is a chance to overcome these difficulties in the future and start to implement hyperpolarization experiments known to work in the X-band regime, such as e.g., NOVEL, the Frequency Swept Integrated (FS-ISE), the Stretched Solid Effect (SSE) also in high-field DNP enhanced NMR.

**Thomas Prisner**, Frankfurt, reported on pulsed EPR techniques for structural studies of flexible groups in biomolecules. After a careful introduction into dipolar EPR spectroscopy, employing techniques like PELDOR (DEER) for distance measurements by measuring the dipolar coupling between unpaired electron spins, he discussed spin-labelling schemes suitable for high-precision distance measurements in nucleic acids (RNA, DNA) and their evaluation by MD simulations and NMR constraints.

**Kazunobu Sato**, Osaka, discussed the potential and application of pulsed EPR techniques in quantum information technology. He reported the manipulation of unpaired electronic spins in molecules, acting as qubits in quantum spin technology, employing a pulsed ESR spectrometer with an arbitrary waveform generator (AWG), that enables the implementation of advanced pulsed ESR experiments including gradients and the manipulation of the states the electronic spins by these sequences.

**Sami Jannin,** Lyon, discussed alternative approaches for dissolution DNP hyperpolarization, where the initially created ^1^H hyperpolarization is transferred to ^13^C-nuclei with very long polarization lifetimes by different CP techniques. The ^13^C-nuclei serve as a means of storage of the hyperpolarization on the order of hours and beyond. To achieve these storage possibilities, they developed special new porous polymeric or silica materials, which can be impregnated with solutions of the hyperpolarization target. The talk introduced the design of these systems and their application in DNP experiments.

**Valentin Novikov**, Moscow, discussed how paramagnetic NMR spectroscopy can be employed to analyze the magnetic properties of molecular systems and reveal possible spin crossover (SCO) or single molecular magnet (SMM) behavior, employing an analysis of the temperature dependence of their chemical shifts. In contrast to conventional magnetometry, the presented paramagnetic NMR approach does not depend on high purification of the material under investigation and is applicable to typical real-world systems, such as e.g., mixtures of different compounds.

**Songi Han**, Berkeley, discussed the role of the electron spin dynamics and of electronic spin couplings for efficient dynamic nuclear polarization enhancement of nuclear spins by means of the Cross Effect (CE) and the Thermal Mixing (TM) process. In both cases, the electronic coupling network provides a means for achieving the necessary energy compensation of the nuclear spin flips. In her presentation, she showed how the TM process can be effectively employed also at very high magnetic fields employing special classes of polarizing agents (Trityl-OX06, BDPA), which tend to aggregate to molecular clusters and form an electron spin coupling network. With these new PAs it was possible to achieve a very high DNP enhancement event at a 900 MHz spectrometer and under fast MAS.

**Sabine van Doorslaer**, Antwerp, reported on recent advances in hyperfine spectroscopy, i.e., EPR techniques revealing the interactions of electronic spin with nuclear spins, such as e.g., ENDOR or ESEEM. In her presentation an overview of the most important hyperfine techniques was given, it was shown how hyperfine spectroscopy profits from the usage of shaped microwave pulses and current challenges in detection and simulation of the hyperfine spectra were discussed.

**Dominik Bucher**, Munich, discussed the usage of optically probed nitrogen-vacancy (NV) point defects in diamonds as a new class of quantum sensors for the very sensitive detection of magnetic fields. After a clear introduction into quantum sensing with NV-centers and detection of NMR signals, recent achievements in hyperpolarized quantum sensing were reported and the application potential of the method in biology and chemistry was discussed.

**Stephen Hill**, NHMFL, gave an exciting overview about recent advances in broadband pulsed EPR spectroscopy, achieved at the NHMFL. After an introduction of the 94 GHz broadband EPR spectrometer, a number of characteristic applications which exploit the broadband capability of the instrument were reported. These examples included spectral hole-burning experiments followed by an ESE detection, via frequency scanning of Fourier transform experiments employing chirped pulses.

**Jörg Matysik**, Leipzig, finally reported the basic foundations and recent progress in the application of the “photo-CIDNP” (photochemically induced dynamic nuclear polarization) effect towards the study of the photosynthetic reaction center. In particular, the origins of the functional symmetry break in purple-bacterial RCs and the high quantum yield of light-driven electron transfer were discussed.

## Organization and Future Developments

The conference was again organized by Konstantin Ivanov (ITC, Novosibirsk, Russia), Gerd Buntkowsky (TU, Darmstadt, Germany), Daniel Abergel (ENS Paris, France), and P. K. Madhu (TIFR Hyderabad, India). Suman Saurav, TIFR Hyderabad, provided technical assistance. The conference and seminar series were sponsored by Alexander von Humboldt Foundation, Wiley, Springer, HyperSpin, and Adani. Owing to the very favorable reactions and in response to the encouraging comments from the MR community, there are already plans to convert ICONS conferences into a regular series of meetings, following the scheme of a general MR conference in summer and a specialized conference on cutting-edge topics in winter. These meetings will be held either purely online or after the end of the current pandemic also as physical meetings. For details and the schedule of upcoming talks see the home page of the meeting ICONS-Seminary.
